# Macroscopic Structural and Connectome Mapping of the Mouse Brain Using Diffusion Magnetic Resonance Imaging

**DOI:** 10.21769/BioProtoc.4221

**Published:** 2021-11-20

**Authors:** Tanzil Mahmud Arefin, Choong Heon Lee, Jordon D. White, Jiangyang Zhang, Arie Kaffman

**Affiliations:** 1 Bernard Irene Schwartz Center for Biomedical Imaging, Department of Radiology, New York University Grossman School of Medicine, New York, USA; 2 Department of Psychiatry, Yale University School of Medicine, New Haven, Connecticut, USA

**Keywords:** Diffusion MRI, Fiber tractography, Structural connectivity, Brain network properties, Mouse brain.

## Abstract

Translational work in rodents elucidates basic mechanisms that drive complex behaviors relevant to psychiatric and neurological conditions. Nonetheless, numerous promising studies in rodents later fail in clinical trials, highlighting the need for improving the translational utility of preclinical studies in rodents. Imaging of small rodents provides an important strategy to address this challenge, as it enables a whole-brain unbiased search for structural and dynamic changes that can be directly compared to human imaging. The functional significance of structural changes identified using imaging can then be further investigated using molecular and genetic tools available for the mouse. Here, we describe a pipeline for unbiased search and characterization of structural changes and network properties, based on diffusion MRI data covering the entire mouse brain at an isotropic resolution of 100 µm. We first used unbiased whole-brain voxel-based analyses to identify volumetric and microstructural alterations in the brain of adult mice exposed to unpredictable postnatal stress (UPS), which is a mouse model of complex early life stress (ELS). Brain regions showing structural abnormalities were used as nodes to generate a grid for assessing structural connectivity and network properties based on graph theory. The technique described here can be broadly applied to understand brain connectivity in other mouse models of human disorders, as well as in genetically modified mouse strains.

Graphic abstract:

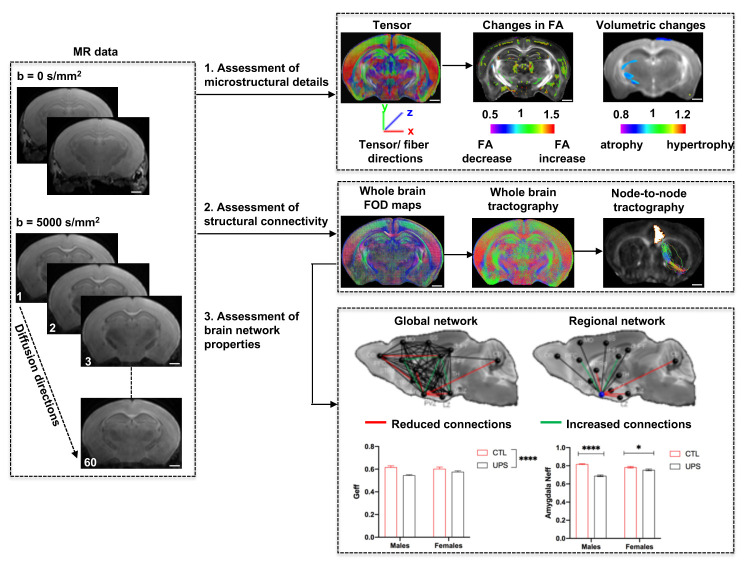

**Pipeline for characterizing structural connectome in the mouse brain using diffusion magnetic resonance imaging.** Scale bar = 1 mm.

## Background

Diffusion magnetic resonance imaging (dMRI) is an imaging technique that uses the random diffusion of water molecules to probe tissue microstructure (Le Bihan, 2003; Mori and Zhang; 2006; Novikov, 2021). Recent advances in imaging and computational processing allowed dMRI images with 100 µm resolution or higher to be obtained from rodents (Aggarwal *et al.*, 2010; Calabrese *et al.*, 2015). These can then be used to assess local volumetric changes through microstructural alterations in dMRI parameters, such as fractional anisotropy (FA), and to determine structural connectivity between different brain regions (Wu *et al.*, 2013; Lerch *et al.*, 2017; Feo and Giove, 2019; Badea *et al.*, 2019; White *et al.*, 2020; Pallast *et al.*, 2020).

High resolution dMRI studies in small rodents provide a novel and promising frontier for improving the translational utility of preclinical studies (Kaffman *et al.*, 2019; Muller *et al.*, 2020). This is primarily because of the direct comparison that can be drawn with parallel studies in humans. In addition, unbiased voxel-based screening can identify specific brain regions that show structural changes. In turn, these can be used as nodes to construct a network and to characterize structural connectivity between specific nodes, identify critical hubs, and quantify network properties, such as global efficiency and small-worldness (Feo and Giove, 2019; Pallast *et al.*, 2020; White *et al.*, 2020). This unbiased agnostic approach is conceptually different from the more traditional region of interest (ROI) approach, in which structural changes in specific brain regions or connectomes are examined (Helmstaedter *et al.*, 2013; Takemura *et al.*, 2013; Saleeba *et al.*, 2019). Nonetheless, dMRI studies are highly complementary with traditional neuroscience approaches, as structural changes identified by dMRI can be further examined using microscopy and genomic/proteomic approaches, and their contribution to complex behavior can be rigorously tested using chemogenetic and optogenetic tools (Kaffman *et al.*, 2019; Muller *et al.*, 2020).

dMRI provides multi-level information about structural changes in the intact tissue, including volumetric changes, dMRI parameters related to microstructure, and structural connectivity. The last fifteen years have witnessed rapid development in dMRI-based tract reconstruction, or tractography (Tuch *et al.*, 2002; Mori and van Zijl, 2002; Tournier *et al.*, 2007; Wedeen *et al.*, 2008), which serves as an important component of the Human Connectome Project (Toga *et al.*, 2012; Van Essen *et al.*, 2013). With the development of high-resolution dMRI acquisition and tractography methods, dMRI tractography can now quickly survey macroscopic structural connectivity in the entire brain without sectioning, which is time consuming and prone to distortions and tissue damage (Moldrich *et al.*, 2010; Wu *et al.*, 2013; Calabrese *et al.*, 2015; Xiong *et al.*, 2018). It also permits simultaneous examination of multiple white matter connections in the same specimen, further reducing the time and cost. With the latest tools for brain connectivity analysis, tractography results can be used to examine changes at both individual pathways and entire connectome levels (Edwards *et al.*, 2020).

dMRI also has several drawbacks, including lower resolution in gray matter regions compared to T1/T2-weighted MRI (Dorr *et al.*, 2008; White *et al.*, 2020) and limited spatial resolution and specificity compared to light microscopy findings with chemical or viral tracers (Wu and Zhang, 2016; Edwards *et al.*, 2020). The need to rigorously correct for multiple comparisons when conducting whole-brain voxel analysis further hinders the detection of subtle changes and is particularly challenging when looking for interaction between two variables, such as early life stress (ELS) and sex (White *et al.*, 2020). High resolution dMRI usually requires perfusing the animal, which prevents longitudinal rescanning of the same animals. Although techniques for *in vivo* high resolution dMRI of rodent brains have emerged (Wu *et al.*, 2013; Wu *et al.*, 2014), the exposure to anesthesia during MRI (2-3 h per session) may introduce additional confounding factors. Therefore, portraying a standardized procedure for reliable and reproducible estimation of microstructural changes in the mouse brain is crucial.

The protocol described here covers image acquisition, whole brain voxel analyses for volumetric and FA changes, tractography, and analysis. Compared to similar methods described before (Calabrese *et al.*, 2015; Edwards *et al.*, 2020), this protocol is based on the structural labels in the Allen Mouse Brain Atlas, which makes it relatively straightforward to compare tractography results with viral tracer results in the Allen Mouse Brain Connectivity Atlas (Oh *et al.*, 2014; White *et al.*, 2020). Unbiased whole-brain voxel analyses were used to identify brain regions that show changes in volume and dMRI parameters (*e.g*., FA) induced by ELS, and to compare them with those reported in humans exposed to early adversity. Fourteen brain regions that showed structural changes were used as nodes to generate a 14 × 14 matrix in each hemisphere. The network properties of this grid were then characterized using graph theory and compared with findings in humans exposed to early adversity (White *et al.*, 2020). Our protocol relies on precise image registration to transfer structural labels from the atlas to subject images and will not work when there are large tissue deformations, such as those caused by brain tumors or severe necrosis. The protocol also has a node-to-node analysis step for small connections (*e.g*., in the amygdala network) that may be obscured in a whole brain analysis. Altogether, the protocol is useful for characterizing whole brain structural connectivity in mouse models of diseases.

## Materials and Reagents

1. 5 ml syringe (Sigma-Aldrich, catalog number: Z683582-100EA)
2. Vacutainer safety-lock blood collection set (25 G × 3/4” × 12”, 0.5 × 19 × 305 mm, Becton Dickinson, catalog number: 367283)
3. Nylon Zip ties (4” and 8” in length, LECO plastics, part# L-4-18, L-8-50)
4. 50 ml conical tubes (Corning, catalog number: 352070)
5. BALB/cByJ mice (Jackson Laboratories, catalog number: 001026, 8-10 weeks old, males and females)
6. Chloral hydrate (Sigma, catalog number: 102425)
7. Heparin (Sigma, catalog number: H3393-50KU)
8. PBS (Corning, catalog number: 21-031)
9. Gadodiamide (Omniscan, CAS# 131410-48-5)
10. 10% Formalin solution (PolyScience, catalog number: 08279-20)
11. Perfluoropolyether (Fomblin^®^, PerkinElmer LLC, CAS# 69991067-9, Sigma-Aldrich, catalog number: 317926)

## Equipment

1. Tools for routine transcardiac perfusion in mice (peristaltic pump and tubing, sharp small scissors, blunt tweezers, 21 G infusion butterfly (Becton Dickinson, catalog number: 367281), large container for blood collection, top of an insulated foam box to pin the mouse, and 23 G needles.
2. Horizontal 7 Tesla (T) Magnetic Resonance (MR) system (Bruker Biospin, Billercia, MA, USA) or other high-field (7T or greater) MRI system
3. 4-channel receive only cryogenic probe (Bruker Biospin, Billerica, MA, USA)
4. 72 mm inner diameter volume transmit coil (Bruker Biospin, Billerica, MA, USA)
5. Animal holder for the cryogenic probe (Bruker Biospin, Billerica, MA, USA)
6. Vacuum and vacuum chamber (*e.g*., 1-gal)

## Software

1. Paravision (PV 6.0.1 or later)
2. Matlab R2019b or later (www.mathworks.com)
3. DTIStudio (www.mristudio.org)
4. AMIRA (thermofisher.com, version 5.0 or later)
5. DiffeoMap (www.mristudio.org) or ANTs (http://stnava.github.io/ANTs/)
6. Mrtrix (www.mrtrix.org)
7. Graph theoretical network analysis toolbox (GRETNA) (www.nitrc.org/projects/gretna)
8. GraphPad Prism (Version 8.4.3 for Windows, GraphPad Software, La Jolla California USA) (www.graphpad.com)

## Procedure

- *Ex-vivo* brain sample preparation
  1. Anesthetize the mouse with chloral hydrate (intraperitoneal injection 100 mg/kg in sterile PBS).
  2. Transcardially perfuse the mouse with 35 ml of cold PBS/heparin (50 units/ml) solution followed by 35 ml of 10% formalin. The perfusion rate is approximately 12 ml/min for PBS and formalin, with good perfusion assessed by the liver changing color from dark red to brownish/grey and the animal carcass becoming stiff.
  3. Decapitate the mouse at the mid-cervical line (around C3-C4), making sure not to damage the spinal cord, and place the head in 50 ml 10% Formalin solution at 4°C for 24 h in a 50 ml conical tube.
  4. After 24 h post-fixation, replace the formalin with PBS. Samples can be stored at 4°C at this point until ready to be scanned.
  5. Replace the PBS solution with 50 ml of 2 mM gadodiamide solution in PBS.
  6. Store the sample at 4°C for one week for the gadodiamide to diffuse into the tissue.
  7. Trim the skin and muscle tissues but keep the skull and eyeballs intact (Figure 1A). Remove the mandible bone and the tongue.
  8. Place one brain in the barrel of a 5 ml syringe, with the nose facing the hub of the syringe, then place 2-3 small pieces of bent zip-tie at the bottom and back of the brain to properly fix the specimen within the syringe barrel (Figure 1A).
  9. Replacing the cap of the syringe with a loosely tied vacutainer, fill the syringe with perfluoropolyether (Fomblin^®^), insert the plunger, flip the syringe so that the hub points upward, and remove the cap.
  10. Place the syringe with its hub pointing upward in a vacuum chamber for 30 min to remove air bubbles (Figure 1B).
  11. Remove the syringe from the vacuum chamber, push out the remaining air and PBS in the barrel, and seal the cap of the syringe by tightening the vacutainer (Figure 1C).
- MR data acquisition
  1. Place the syringe horizontally in the animal holder for the cryogenic probe and adjust the sample position so that the dorsal part of the brain is as close to the cryogenic coil as possible to maximize sensitivity. Use tape to fix the syringe to the animal holder (Figure 1D).
  2. Insert the animal holder into the magnet under the cryogenic probe (Figure 1E).
  3. Acquire a pilot scan using the Bruker Localizer protocol. For any MRI studies, Localizer is the very first scan that acquires reference images of the subject in three orthogonal planes. The images of the resulting scan appear in the ‘geometry editor,’ where the first three viewports show the reference brain slices in axial, sagittal, and coronal orientations. Therefore, the Localizer provides a quick view of the specimen in the magnet (Figure 1F). Check whether the sample is in the most sensitive region of the cryogenic probe with no apparent tilt toward the left or right sides. Adjust the position of the subject and re-run the Localizer protocol prior to proceeding to the next step.
  4. Adjust the tuning and match of the cryogenic probe and acquire a map of the main magnetic field (B_0_ field) over the entire sample.
  5. Use the Bruker MapShim procedure to adjust shimming currents to achieve a relatively homogeneous B_0_ field. In brief, select the specific scan to calculate the shim. Then choose Map_shim from the setup tab and define the target volume of interest in cubic, cylinder, or ellipsoid shapes. Shift, resize or rotate the target volume in the geometry editor such that the volume covers the entire specimen. Run the scan to compute the optimum shim values in the target volume based on the B_0 _map measured in the previous step.
  6. Acquire high angular resolution diffusion weighted imaging (HARDI) of the whole mouse brain using a modified 3D gradient and spin echo (GRASE) sequence (Wu et al., 2013) (an alternative is the 3D multi-shot diffusion weighted echo planar imaging (EPI) sequence provided by Bruker) and with the following imaging parameters:
    - Echo time (TE)/repetition time (TR): 33/400 min.
    - Number of non-diffusion weighted images (b_0_s): 2.
    - Number of diffusion weighted images (DWIs): 60, auto-generated by the sequence.
    - b-value: 5,000 s/mm^2^.
    - Resolution: 100 µm isotropic.
  **Figure 1.**
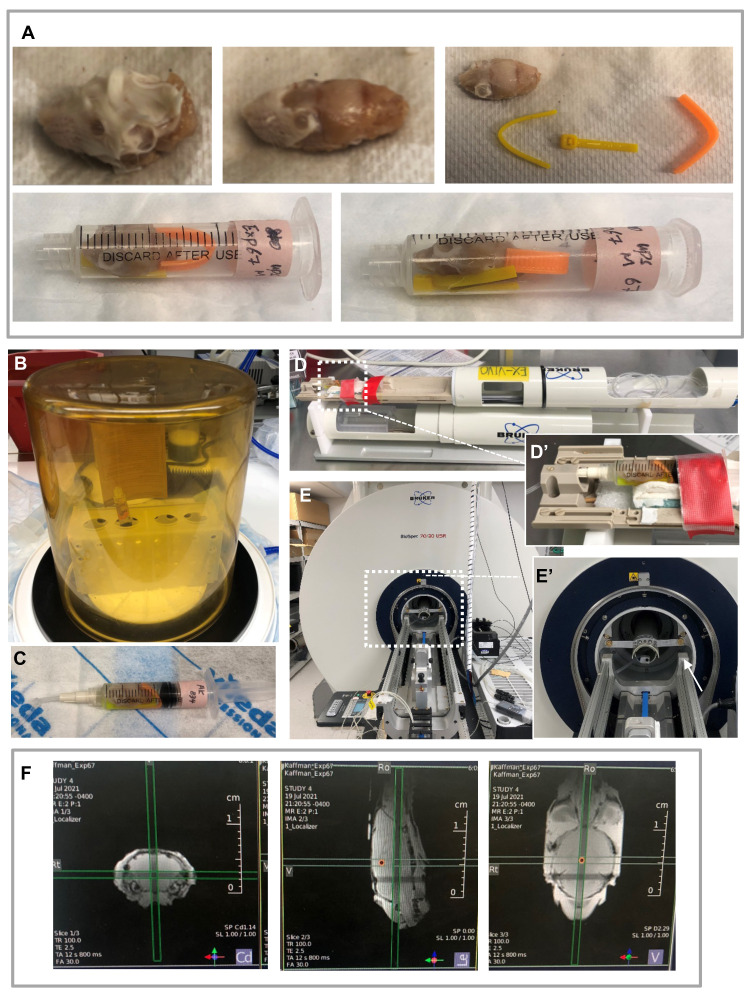
Preparation for MRI. A. Sample preparation: Remove the tissues outside of the skull carefully without damaging the eyeballs (top panel). Place the brain in a 5ml syringe with small pieces of zip-ties to fix its position (bottom panel). B-C. Remove air from the syringe: Connect the syringe to a loosely tied vacutainer filled with Fomblin^®^ (shown in C) and place in the vacuum chamber (shown in B). Remove the vacutainer and turn on the vacuum for 30 min to remove air bubbles. Push out the remaining air after vacuum and seal the top by tightening the vacutainer. D. Place the sample in a manufacturer-made sample holder designed for the cryogenic probe. D’. A zoom-in view of the sample. E. Insert the sample holder into the magnet bore of the magnet. E’. A zoom-in view of the holder (indicated by the white arrow). F. Three orthogonal plane images acquired using the Localizer protocol on a 7 Tesla Bruker preclinical MRI system.

## Data analysis

- Data pre-processing
  For each dataset, perform the following steps accordingly:
  1. Motion correction: Using DTIStudio (Jiang *et al.*, 2006), align all DWIs to the average of b_0_s to remove small sample displacements due to vibrations and B_0_ field drift during the long scan (Figure 2A).
  2. Skull-stripping: Use AMIRA segmentation editor to remove non-brain tissues and define the subject specific whole brain mask (Figure 2B).
  3. Estimation of diffusion tensor: From the raw data, compute the tensor model (Mori and Zhang, 2006) within the respective brain mask using weighted linear least squares estimations as implemented in MRtrix (command: dwi2tensor) (Figure 2C) (Tournier *et al.*, 2012).
  4. Computation of average DWIs and fractional anisotropy (FA): Compute the average DWI (aDWI) from 60 DWIs using Matlab, and calculate the FA map from the tensor using MRtrix (command: tensor2metric) (Basser *et al.*, 1994; Tournier *et al.*, 2012).
  5. Image registration and transfer of atlas labels into subject’s native space: Using DiffeoMap, normalize the aDWI and FA maps to an MRI-based atlas (Chuang *et al.*, 2011; Arefin *et al.*, 2019) via multi-channel (aDWI + FA) large deformation diffeomorphic metric mapping (LDDMM) (Ceritoglu *et al.*, 2009) (Figure 3A). Next, transfer the structural labels (*i.e*., brain regions or nodes) to the subject’s-native space using the inverse mapping from LDDMM (Figure 3B, also see Note 1). If DiffeoMap is not available, ANTs (http://stnava.github.io/ANTs/) can be used instead (Figure 2D).
    **Figure 2.**
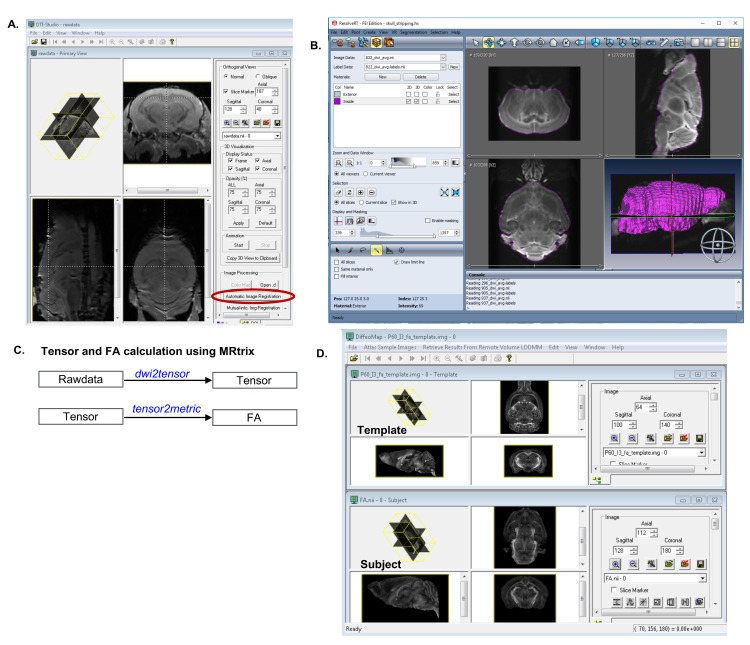
Illustration of the data pre-processing steps. A. Motion correction using DTIStudio. Run Automatic Image Registration (circled) to align all diffusion weighted images (DWIs) to the non-diffusion-weighted image (b_0_). B. Use the AMIRA segmentation editor to generate a binary mask (purple) for the brain. C. Schematic diagram of the steps to compute the tensor and FA from the rawdata using Mrtrix. D. Use DiffeoMap for image registration and transformation of atlas labels into subject’s native image space.
    **Figure 3.**
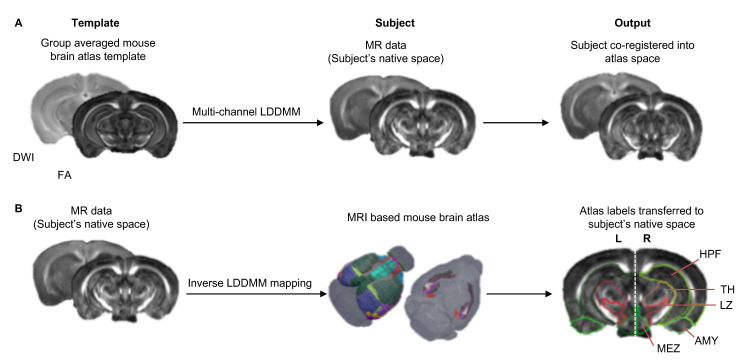
Image registration pipeline. A. Co-registration of the MR data (subject) into group averaged mouse brain atlas template using multi-channel LDDMM. B. Transformation of structural labels from MRI-based atlas to subject’s native space.
- Data post-processing
***Assessment of brain microstructural changes***
At first, compute the Jacobian determinant value for each voxel from the mapping between atlas and subject images generated by LDDMM, and conduct whole-brain voxel-based morphometric analysis in Matlab to identify local volumetric changes affected by rearing, sex, and their interaction (2 × 2 ANOVA, FDR corrected, α = 0.1, *P* < 0.0105, cluster size > 25 voxels). See the Matlab codes (source data 1) used to conduct 2 × 2 ANOVA (White *et al.*, 2020). Then, similarly perform 2 × 2 ANOVA (FDR corrected, α = 0.1, *P* < 0.007, cluster size > 25 voxels) to examine the voxel-wise changes in FA (White *et al.*, 2020). These analyses will provide unbiased overviews of morphometric changes due to rearing, sex, and rearing by sex interaction.
***Selection of brain regions (nodes) for structural connectivity assessment***
  Identify nodes that show rearing-mediated volumetric and FA changes to investigate structural connectivity alterations between nodes, as well as modifications in the brain global and regional network properties (also see Note 2). These nodes will be identical for both left and right hemispheres.
***Assessment of brain structural connectivity using fiber tractography***
  Upon pre-processing the data and selection of potential brain nodes, execute the following steps accordingly for each individual subject to map axonal projections between nodes using probabilistic fiber tractography in MRtrix:
Step 1: From the pre-processed raw data, estimate the response function for spherical deconvolution (command: *dwi2response*) (Tournier et al., 2012, Tournier et al., 2013). Specify the algorithm name ‘tournier’ (other options: dhollander, manual, fa, msmt_5tt, tax), gradient table, brain mask, and the maximum harmonic degree (l_max _= 6).
Step 2: Estimate the whole brain fiber orientation distribution (FOD) map from the pre-processed raw data and respective response function (command: *dwi2fod*) (Tournier et al., 2007). Define the algorithm name ‘CSD,’ gradient table, and brain mask.
Step 3. Generate the whole brain fiber tractogram from the FOD map (command: *tckgen*) (Tournier et al., 2009). Use the whole brain mask as the ‘seed region’ to enable tracking fibers throughout the brain for whole brain tractography (whole brain tractogram) (Figure 4A). Set the tractography method to probabilistic, the FOD amplitude cut-off to 0.05, the minimum length of the fiber to 3 mm, and the target number of the streamlines to be counted to 5 million.
Step 4: For node-to-node tractography, the whole brain tractography in step 3 may not generate enough streamlines for small nodes (*e.g*., amygdala). Further increasing the total number of streamlines (> 5 million) may not resolve this issue but requires significant computational resources. In this case, extract the regions of interest (ROIs) from the atlas co-registered into the subject’s native space using Matlab. Next, define a specific node as ‘seed region’ to initiate the fiber tracking from and another node as ‘target’ to define the fiber termination point. Then use these two nodes to extract the streamlines connecting two nodes (seed and target) using the *tckedit* command (Figure 4B). Consider two nodes as ‘connected’ if there is at least one streamline terminating at the target node; otherwise, they are ‘not connected.’
  **Figure 4.**
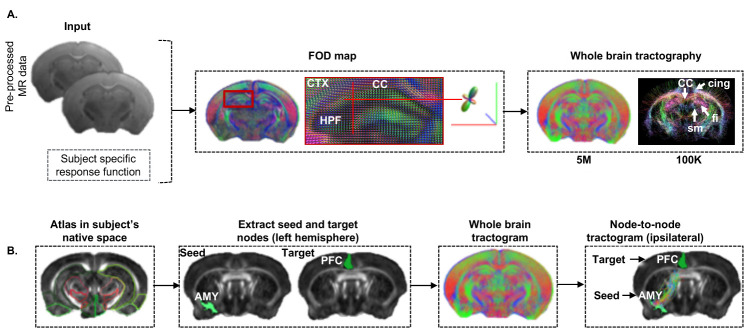
Fiber tractography pipeline. A. Estimation of mouse whole brain fiber tractogram from the fiber orientation distribution (FOD) map. Red, green, and blue colors represent the fiber projections in x, y, and z-axis, respectively. Five million fibers were generated from each subject; 100 K streamlines were extracted for better visualization of the brain structures. B. Extraction of fibers connecting two specific nodes (seed = amygdala and target = PFC).
***Generating brain structural connectome matrix***
Repeat step 4 to estimate the structural connections between all possible pairs of nodes (ignore intra-regional connectivity) for both hemispheres (Figure 5A). For example, for 14 nodes in one hemisphere, the total number of tractograms would be the number of nodes N = 14 multiplied by N-1, or 14 × 13 = 182. Finally, for *M* number of seed regions and *N* number of target regions, generate an *M* × *N* matrix individually for the left and the right hemispheres. Assign the seed and target regions in horizontal and vertical axis, respectively, so that each cell represents the number of streamlines connecting the corresponding seed and target nodes (Figure 5B). Consider the number of streamlines between nodes as a measure of the connection strength. Generate the connectome matrix for all subjects and name them according to the subject IDs.
  **Figure 5.**
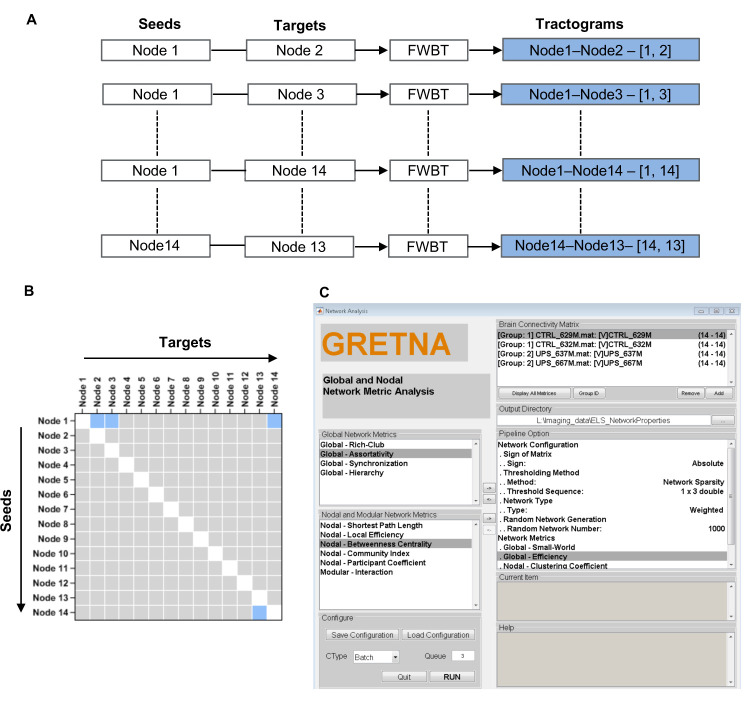
Generation of the mouse brain structural connectome. A. Extraction of fibers connecting seed and target nodes. B. Generation of structural connectome from the tractograms estimated from selected seed and target nodes. Blue cells correspond to the tractograms shown in A, and white cells indicate intra-regional connectivity (not counted). C. Use the GRETNA software to compute global and regional brain network properties. Panels on the left list all possible properties available for computation. Select the properties based on the study design and transfer them to the pipeline option on the right panel using the respective arrows. Load the connectome matrix for all subjects belonging to one group with specific group ID and then load for the next group with different ID. Specify the output folder to store the results and define the network configuration. Finally, hit the ‘Run’ button to start computation.
***Brain network properties analysis***
Use the Matlab based Graph theoretical network analysis toolbox (GRETNA) to compute the brain global and regional network properties (Wang *et al.*, 2015). Perform the following steps accordingly for brain network-based analysis (Figure 5C):
  1. Create an individual data folder containing two sub-folders for left and right hemispheres for each group.
  2. Save the connectome matrices as ‘.*mat*’ files in the respective folders.
  3. Open GRETNA in Matlab and select ‘*Network Analysis*’ (GRETNA >> Network Analysis).
  4. In the ‘*Brain Connectivity Matrix*’ tab, load all connectivity matrices of one hemisphere from one group and assign the group ID. Do the same for the other group.
  5. Locate a directory for saving the results in the ‘*output directory*’ tab.
  6. Next, select network properties to be computed from the Global Network Metrics and Nodal and Modular Network Metrics tabs.
  7. For global brain network analysis, select *‘Global – Small-World (SW)’* and *‘Global – Efficiency (Geff).’* For regional network properties, select ‘*Nodal – Clustering Coefficient (NCp),’ ‘Nodal – Efficiency (Neff)*,’ and ‘*Nodal – Degree Centrality (Dcent)*.’ Other properties can be selected as per the study design or requirements.
  8. Configure the brain network in the ‘*Network Analysis*’ tab as follows:
**Parameters Value**
    Sign of matrix Absolute
    Thresholding method Network sparsity
    Threshold sequence 0.05, 0.1, 0.15 (or as per the study design)
    Network type Weighted
    Random network number 1,000
  9. Recheck the loaded data and the network configuration. Hit the ‘*Run*’ button if everything looks alright. Computation time depends on the number of subjects, size of the connectome matrices, random network number, and the threshold sequence.
  10. Once the computation is done, results can be retrieved from the output directory. For further assistance, please refer to the following manual from Neuroimaging Tools and Resources Collaboratory (NITRC): https://www.nitrc.org/docman/view.php/668/2262/manual_v2.0.0.pdf.
***Statistical analysis of the estimated structural connectivity and brain network properties***
  To investigate the effect of rearing and sex on brain structural connectivity and brain network properties, perform a two-way ANOVA with rearing condition (CTL or UPS) and sex as fixed factors, followed by post-hoc comparisons using Tukey’s HSD or Sidak’s test using GraphPad Prism.

## Notes

1. It is very important to check whether structural labels were correctly transferred and show good agreement with the corresponding structures. We recommend refining the segmentation manually, slice by slice, along the axial orientation, forfeiting attention to the other two orientations as well as to the slices preceding and following if necessary.
2. Selection of nodes for brain network analysis is crucial. Using unbiased voxel-based analyses, identify only those nodes which show UPS-mediated volumetric and FA alterations and are highly connected based on the Allen Mouse Brain Connectivity Atlas (Oh *et al.*, 2014). Furthermore, selected nodes should be non-overlapping, having a unique set of connections to other nodes, and well delineated using a standard parcellation scheme that is comparable across species (Kaiser, 2011).
